# Facts for the People

**Published:** 1848-04

**Authors:** H. Crane


### FACTS FOR THE PEOPLE—CONTINUED.

BY H. CRANE, D. D. S.

"One of the leading causes of the destruction of the human tooth, is, in my humble opinion, the rapid physical degeneracy of the human species.

"Your manifestation of surprise, madam, will not alter the fact. The teeth are parts and parcels of the human frame, highly endowed with the functions of life, and governed in all its interminable minutia by its laws, and all deterioration from a full and perfect development of physical man, impairs this function, thereby rendering that life which is derived from life, infirm, and incapable of fulfilling the great mission for which it was created.

"In proof of my position, you may recur to the universal principle of life, and behold, both in the animal and vegetable kingdom, the same general laws, fixed and irrevocable.

"Tell me when and where it has been customary to select the delicate plant, from which to produce healthy and vigorous fruit. Mahon tells us "that man cannot be born with a strong constitution, unless he derives his life from parents who are in perfect health, and in the stıength of age."

"He further says, 'that a child born of weakly parents may become strong; but that it will always present certain signs of its primedial constitution.' But there are other causes operating, which result in the destruction of the human tooth, some of which I shall briefly allude to. Teeth are often lost for want of cleanliness. Alimentary particles lodge between the teeth, which if permitted to remain, become active agents in hastening their destruction. "Chemistry teaches us that all vegetable or animal substances, in a state of decomposition, give birth to acidiferous products, producing the same effect as caries upon the teeth, hence the necessity of using the brush daily. Acids and warm drinks, are among the local causes which produce caries.

"There are as many causes of dental caries, as there are remedies for the tooth-ache. Consequently, it would be folly for me to attempt to do the subject justice in this place. Suffice it to add, that all bone, muscles, and I might also include brains, become enfeebled, and short lived in the same ratio that habits of ease and inactivity are contracted. Madam, have I answered your question?"

"Doctor, do you intend to convey the idea that the sedentary habits and mode of living adopted by the present generation, and particularly the female portion of it, are unfavorable to a good constitution, or good teeth?"

"Most undoubtedly, madam. The cities are the hot houses, and the grave yards of the country, morally and physically, and the country is to some extent, copying their example. Little feet, tapering fingers, and pale faces are all the go, and foul breath, and dirty teeth follow."

"George has four of his last molar teeth, and as they will never be renewed, it will be necessary to have them preserved—two of them require filling."

"What do you mean by 'last molar teeth,' doctor, George is but seven years old and he never has lost any of his double teeth."

"Very true, madam. There are eight molar teeth, and four wisdom teeth that never appear a second time, consequently, you must watch them with great caution, and have them plugged immediately on the appearance of disease, if you desire to save them."

"Doctor, do you observe those tusks in George's mouth?"

"They are not tusks, madam, but his eye teeth, the strongest and most valuable in the set. They are forced into that position by the small double teeth appearing before them, and the arch of the jaw not being sufficiently large to receive both. The two small double teeth immediately behind the eye teeth should be removed to save deformity."

"But they are the last teeth, doctor."

"True, they are the last teeth, but the arch will be complete without them, and both will ultimately be lost if they are permitted to remain so crowded and irregular,—for you are probably aware, it is the very next thing to an impossibility, to save teeth permanently by plugging, while in such chaos, and besides they are much more liable to decay."

"You allude to plugging teeth, I have lost all confidence in the declaration made by dentists, 'that all teeth can be saved by plugging.' My own practice proves the contrary."

"It is true that a large majority of plugs inserted, are valueless, and put in by men who either know but little about this branch of the business, or who possess too little principle to do justice to their patients, to themselves, or to the profession."

"Scarcely a paper appears in the city, that does not contain the advertisements of some of the scabs of the prfession, proposing to perform operations, "all warranted," at less price than the cost of good materials. The people are humbugged by this stratagem, and besides loosing their money, loose their confidence in the whole fraternity. The plugs either "come out," or if they remain, the tooth becomes discolored and finally lost, through this species of rascality."

